# Reliability and validity of a new scale on internal coherence (ICS) of cancer patients

**DOI:** 10.1186/1477-7525-7-59

**Published:** 2009-06-24

**Authors:** Matthias Kröz, Arndt Büssing, Hans Broder von Laue, Marcus Reif, Gene Feder, Friedemann Schad, Matthias Girke, Harald Matthes

**Affiliations:** 1 Havelhöhe Research Institute (FIH) at the Community Hospital Havelhöhe, Kladower Damm 221, 14089 Berlin, Germany; 2 Department of Internal Medicine, Community Hospital Havelhöhe, Kladower Damm 221, 14089 Berlin, Germany; 3 Chair of Medical Theory and Complementary Medicine, University of Witten/Herdecke, Gerhard-Kienle-Weg 4, 58313 Herdecke, Germany; 4 Oncological Practice, Öschelbronn, Am Eichhof 30, 75223 Niefern-Öschelbronn, Germany; 5 Institute for Clinical Research (IKF), Hardenbergstr. 19, 10623 Berlin Germany; 6 Unit of academic primary health care, Bristol University, 25 Belgrave Road Bristol BS8 2AA, UK

## Abstract

**Background:**

Current inventories on quality of life used in oncology mainly focus on functional aspects of patients in the context of disease adaption and treatments (side) effects (EORTC QLQ C30) or generically the status of common functions (Medical Outcome Study SF 36). Beyond circumscribed dimensions of quality of life (i.e., physical, emotional, social, cognitive etc.), there is a lack of inventories which also address other relevant dimensions such as the 'sense of coherence' (SOC) in cancer patients. SOC is important because of its potential prognostic relevance in cancer patients, but the current SOC scale has mainly been validated for psychiatric and psychosomatic patients. Our two-step validation study addresses the internal coherence (ICS) scale, which is based on expert rating, using specific items for oncological patients, with respect to its reliability, validity and sensitivity to chemotherapy.

**Methods:**

The items were tested on 114 participants (57 cancer patients and a matched control group), alongside questions on autonomic regulation (aR), the Hospital Anxiety and Depression Scale (HADS), self-regulation (SRQ) and Karnofsky the Performance-Index (KPI). A retest of 65 participants was carried out after a median time span of four weeks.

In the second part of the study, the ICS was used to assess internal coherence during chemotherapy in 25 patients with colorectal carcinoma (CRC) and 17 breast cancer patients. ICS was recorded before, during and 4 – 8 weeks after treatment.

**Results:**

The 10-item scale of 'internal coherence' (ICS) shows good to very good reliability: Cronbach-α r = 0.91, retest-reliability r = 0.80. The ICS correlates with r = 0.43 – 0.72 to the convergence criteria (all p < 0.001). We are able to show decreased ICS-values after the third cycle for CRC and breast cancer patients, with a subsequent increase of ICS scores after the end of chemotherapy.

**Conclusion:**

The ICS has good to very good reliability, validity and sensitivity to chemotherapy.

## Background

Since quality of life has gained increasing importance in clinical oncology over the last 15 years, current quality of life inventories now focus on physical, mental, cognitive, social and other dimensions along with chemotherapeutical effects. These comprise the EORTC QLQ C30 [1] of the European Organisation for Research on Treatment of Cancer, including additional scales for specific types of tumours [2] or the Functional Assessment of Cancer Therapy – FACT [3] which, next to the basic module, also contains symptom-specific supplementary modules [4]. Generic questionnaires such as the Medical Outcome Study SF-36, which is also used for oncologic patients, capture the rehabilitative, physical, emotional and social functional status [5].

New studies have yielded some evidence that questionnaires capturing the individual skills of adaptation such as the 'sense of coherence' [6] or 'self-regulation' (Grossarth-Maticek 1999) could be more appropriate as prognostic tools in oncology or in other chronic conditions than conventional health-related quality of life (HRQL) scales [7]. However, inventories which capture the 'Sense of Coherence' (SOC) based on Antonovsky's concept of salutogenesis have mainly been validated in psychosomatic and psychiatric patients [6,8]. Antonovsky's initial question was: What keeps a person healthy? For Antonovsky, health is a continuum between total disease (dis-ease) and complete health (ease). Salutogenesis is a concept based around stress and resilience. The question of salutogenesis contrasts with a central question of scientific medicine into pathogenesis: What makes a person ill? [6]. The SOC is based on three components which are considered prerequisites for salutogenesis: comprehensibility, meaningfulness and manageability.

1) According to Antonovsky, these three factors determine to what extent a person can rely an existing, enduring but also dynamic feeling of trust, which gives structure to the course of internal as well as external events and makes them understandable.

2) These resources have to be available when required and

3) the situation must be understood as challenges which deserve rising to [9].

New epidemiological studies have articulated the potential prognostic relevance of SOC for cancer patients [7]. However, Antonovsky was a sociologist and did not extend his work to people with chronic illness, focusing instead on survivors of the Holocaust. Thus, although the current SOC scale includes general questions about life style, attitudes to life, and mental health [10] which are relevant to all groups of people, it does not address physical health. But rather than containing references to physiological parameters as postulated by Antonovsky [8,9], due to its origin, it focuses in particular on mental health. Already, in 1923 a first medical approach to salutogenesis was discussed [11]. All in all, it therefore there is a need to develop a questionnaire with a stronger focus on patients with chronic diseases, particularly cancer.

Here we report a two stage validity study which aimed to validate the ICS which was developed based on a non-standardised open questioning process of cancer patients before starting the study and expert ratings from questions on disease management, outlook on life and drive, perception of health and thermoregulation for oncological patients. We followed development of the instrument by testing reliability, and validity of construct and content, as well as its responsiveness to chemotherapy.

## Methods

On the basis of a former non-standardised questioning process in 2001 in our centre of tumour therapy, cancer patients described the following symptoms as their major complaints under chemo- and radiotherapy: 1) fatigue, 2) lack of motivation, 3) asthenia, 4) sleepiness, 5) lack of concentration and 6) cogitation, 7) disturbance of memory, 8) depressive symptoms, 9) visual, 10) acoustic, 11) olfactory and 12) gustatory hypersensitivity, 13) feeling indifferent, 14) feeling being not really present, 15) feeling not being coherent, 16) feeling discouraged, .17) feeling uncomfortable, 18) doing activities in a "mechanical manner", 19) having cold hands and feet, 20) often and quick freezing. The symptoms 1 – 7 were integrated in the parallel development process in the CFS-D [19], the symptoms 9–13 have been integrated in the questionnaire as single items.

Based on the symptoms 13 – 20, a group of experts (oncologist, internist, gastroenterologist, somnologist, general practitioner and statistician) formulated twelve items on the topics of meaningfulness, manageability and resilience in dealing with cancer disease and formation of perspective. According to our experience in cancer patients, we also integrated items on heat regulation, because a feeling of comfort and well-being is often associated with good thermoregulation being a precondition on the subject of inner coherence.

### Study 1: item detection, reliability and validity

We carried out this study in the Departments of General Internal Medicine and Gastroenterology at the Gemeinschaftskrankenhaus Havelhöhe (Berlin), as well as in the specialist oncology practice at the hospital between January 2003 and February 2004.

Consecutively recruited patients with histologically confirmed malignant tumours were matched according to age (± 5 years) and gender with participants of a healthy control group who had no known acute or chronic disease. Table 1 shows the demographic, table 2 the clinical and treatment characteristics of the participants. Participants with malignancies had a broad range of tumour localisations (table 3). At the time of being surveyed, at least two weeks had elapsed since a participant's last operation, chemotherapy or radiotherapy session. Further exclusion criteria were patients with a Karnofsky's Index (KPI) < 50%, patients aged < 18 years or > 85 years, patients with a manifest psychosis or uncontrollable pain.

**Table 1:** Sociodemographic overview of the participants of studies 1 and 2

		**Study 1**		**Study 2**	
		
		**Cancer**	**Control**	**BC 1**	**CRC 3**
**Age (y)**	mean ± SD	58.8 ± 11.6	59.9 ± 10.3	54.3 ± 11.6	62.4 ± 10.6
	min/max	30/83	32/81	37/71	33/76
**Gender**	women	41	41	17	13
	men	16	16	0	12
**Marital status**	married	41	20	4	17
	stable partnership	2	4	4	3
	single	2	6	2	1
	divorced	7	11	4	1
	widowed	5	6	1	3
	missing	0	10	2	0

**Table 2:** Overview of tumour stage, diagnosis duration, Karnofsky performance index, haemoglobin and therapy

	**Study 1: Cancer group**	**Study 2:**	**BC**	**CRC**
**Metastasis or generalised stage**				
**yes**	35 (61.4%)		2	3
**No**	24 (38.6%)	Stage 1	3	0
		Stage 2	8	2
		Stage 3	4	18
		missing	0	2

**Initial diagnosis (years)**		**Initial diagnosis (months)**		
Mean (SD)	2.9 (3.1)		1.3 (1.0)	1.4 (0.5)
Median	2.0		1.0	1.0
Min/Max	0/12		1/5	1/2

**Karnofsky-Index (%)**				
Mean (SD)	81.8 (11.6)		86.7 (10.5)	88.7 (9.2)
Median	90		90	90

**Haemoglobin (gr/dl)**				
Mean (SD)	12.1 (1.84)		13.0 (1.28)	13.7 (6.8)
Median	12.5		13.2	12.65
Anemia (<12)	22 (38.5%)		2 (11.1%)	9 (36.0%)

Therapies at the point of questioning		Therapies after first questioning		

**Operation because of cancer**	39 (68.4%)		14 (82.3%)	25 (100%)
**Chemotherapy**	31 (54.4%)		17 (100%)	25 (100%)
**Chemotherapy Scheme**				
CMF+RAD			2	
EC or EC+RAD			7	
EC/CMF or EC/CMF +RAD			3	
FEC or FEC + RAD			1	
EC/Tac			2	
Tac/other			2	
5FU (Mayo mod/Ardalan)				5
5FU + RAD				5
FOLFOX				12
FOLFOX+RAD				1
OXALI mono or others				2
**Radiotherapy**	14 (24.6%)		6 (35.2%)	7 (28.0%)
**Antihormonal therapy**	13 (22.8%)		6 (35.2%)	0 (0)
**Mistletoe therapy**	57 (100%)		17 (100)	25 (100)

**Table 3:** Diagnosis of cancer patients in study 1

**Included**	**N**	**%**
B-chronic lymphatic leukaemia (B-CLL)	1	1.8
B-cell Lymphoma (NHL)	2	3.5
Breast cancer	16	28.1
Bronchial carcinoma (NSCLC)	5	8.8
Carcinoma unclear primary (CUP)	1	1.8
Cervix carcinoma	1	1.8
Colon carcinoma	4	7.0
Gallbladder carcinoma	1	1.8
Hypernephroma	1	1.8
Leiomyosarcoma	1	1.8
Melanoma	1	1.8
Ovarian carcinoma	2	3.5
Ovarian sarcoma	1	1.8
Pancreatic cancer	2	3.5
Pharyngeal cancer	1	1.8
Plasmocytoma	4	7.0
Pleural mesotelioma	3	5.3
Prostatic cancer	3	5.3
Rectum carcinoma	2	3.5
Thymic carcinoma	1	1.8
Thyroid carcinoma	2	3.5
Tonsillar carcinoma	1	1.8
Urethral cancer	1	1.8

**Total**	**57**	**100.0**

Participants in the control group were recruited opportunistically from hospital staff and their families. Any history of malignancy or severe chronic conditions was a criterion for exclusion.

After the study was explained and consent was obtained, the ICS questionnaire was administered. The target for the test-retest analysis was at least 50% of all participants. The questionnaire was re-administered opportunistically without rejection on 65 participants (57.3%) after a median of four weeks (mean of 5.2 weeks, SD = 4.2).

Next to the 12 ICS items (table 4) the following questionnaires were conducted:

**Table 4:** Items of the ICS

**Item**	**Answer Scores**	**Mean values ± SD**	**Item-Total Correlation**	**Factor 1** **Inner Resilience & Coherence**	**Factor 2** **Thermo Coherence**	**Alpha if item deleted**	**Item-Scale Correlation Self Regulation**
1) There were times last week when I felt good	5 - 1	3.55 ± 1.04	0.774	**0.724**	0.415	0.889	0.531
2) I felt cold without reason. *(inverse focusing)*	1 – 5	4.21 ± 1.05	0.527	0.156	**0.897**	0.905	0.312
3) I felt pleasantly warm	5 - 1	3.91 ± 1.13	0.579	0.219	**0.892**	0.902	0.372
4) I felt my health was...	5 - 1	3.30 ± 1.07	0.727	**0.642**	0.478	0.892	0.498
5) I was able to face the day with confidence.	5 - 1	3.80 ± 1.75	0.816	**0.860**	0.269	0.886	0.664
6) I felt confident enough to solve problems in my daily life.	1 – 5	3.86 ± 1.00	0.594	**0.580**	0.335	0.900	0.322
7) I came up with good ways of solving new problems	1 – 5	3.67 ± 0.94	0.569	**0.728**	0.051	0.902	0.575
8) What I did every day was consistent with my inner wishes	5 - 1	3.38 ± 1.09	0.719	**0.769**	0.253	0.892	0.566
9) Deep down I felt secure.	1 – 5	3.68 ± 0.98	0.753	**0.843**	0.196	0.891	0.604
10) I felt I was moving in the right direction.	5 - 1	3.90 ± 0.87	0.585	**0.712**	0.106	0.901	0.532

**Score**	**Sum Scale**			**Inner R. & C**.	**Thermo C**.		**0,661**
**Cronbach-α**	**r_α _= 0.91**			**r_α _= 0.91**	**r_α _= 0.85**		
**Retest-Reliability**	**r_rt _= 0.80**			**r_rt _= 0.74**	**r_rt _= 0.54**		

Answer possibilities with 1: low ICS, 5: high ICS, mean values, item-total-Correlation, principal component analysis result with factor 1: Inner Resilience and Coherence and factor 2: Thermo Coherence, Cronbach-α, test-retest-reliability, item/scale-correlation with self-regulation.

1) The short questionnaire on 'self-regulation' (SRQ) is a scale with 16 items in two subscales for measuring self-regulation and health-building activity with a six-point-Likert scale. The 16 items are added and divided by 16 to obtain a total score. Subscale 1 is termed "Ability to Change Behaviour in order to reach goals", and subscale 2 "Achieve Satisfaction and Well-Being", which thus has a hedonistic connotation. Higher scores indicate better self-regulation. The validity and reliability of the sum and subscales are satisfactory until very good: Cronbach-alpha = 0.80 – 0.95 (and test-retest reliability = 0.73 – 0.82). We used this scale because of its conceptual congruence (convergent validity) with aspects of internal coherence [12,13]

2) The long version questionnaire on 'autonomic regulation' (aR) measures the autonomic functioning of an individual with an 18-item scale in three subscales (orthostatic-circulatory, rest/activity regulation and digestive). AR measures with a three point Likert-scale (18 – 54) with a satisfactory/good reliability and good validity [14,15]. Questions on thermoregulation are integrated into this scale, so we used it to measure convergent validity of items concerning heat regulation [15].

3) The German version of the 'Hospital Anxiety and Depression Scale' (HADS-D) consists of 14 items (7 for anxiety and 7 for depression) on which people rate on a four-point Likert scale (0–21 both). Higher scoring indicate more symptoms, ≥ 11 points anxiety or depression are probable, ≥ 8 – 10 possible cases, < 7 no cases. The HADS is highly reliable and valid and is an extensively used scale in internal medicine research [16,17].

4) The Karnofsky Performance-Index (KPI) [18] which is a general and robust indicator of physical functioning daily life.

5) Questions on autonomic state and heat sensitivity were also collected [15].

Results of the co-validation procedure of the German version of the Cancer Fatigue Scale (CFS-D) have been published elsewhere [19].

Moreover, we documented the last haemoglobin level in the blood (g/dl) before inclusion of all tumour patients. A retest was carried out on 65 participants after a median of 4 weeks.

### Study 2: ICS responsiveness for chemotherapy treatment

From April 2003 to March 2007, the Centre for Tumour Therapy at the Gemeinschaftskrankenhaus Havelhöhe, the oncological practice Öschelbronn and the oncological practice Havelhöhe carried out study 2 to capture responsiveness of the ICS questionnaire to chemotherapy treatment. Consecutively recruited breast cancer and colorectal carcinoma (CRC) patients were examined before or during adjuvant or palliative chemotherapy with mistletoe therapy examining ICS responsiveness.

The following groups were formed in the process:

1) Breast cancer patients who received adjuvant complementary treatment with standardized and commercially available whole plant extracts from Viscum album L. (mistletoe) as standard therapy from the first cycle of chemotherapy (B) or

2) The colorectal cancer (CRC) group (C) was also surveyed prior to the first cycle of chemotherapy with adjuvant mistletoe therapy.

After explanation of the procedure and obtaining informed consent, the following criteria were applied to both groups for adjuvant or initially curatively conceived or palliative chemotherapy with good general condition and a KPI ≥ 70%. The oncologically scheduled chemotherapy, radiotherapy or mistletoe therapy was not impacted or changed by the study so that this second part of the validation study only measured the changing sensitivity (responsiveness) of the instruments. Thus, study 2 is not a pharmacological study to test effectiveness of the treatment which was administered as a routine procedure, but as a sensitivity testing of the instrument.

For this purpose, the surveys were carried out 1 – 5 days prior to the start of chemotherapy (B, C), 1 – 5 days after the third cycle as well as 4 – 8 weeks after the end of chemotherapy using the ICS, CFS-D [19], State autonomic regulation aR [20] and EORTC QLQC30 [1]. For KPI, stage of cancer, surgery and chemotherapy see table 2. The data on CFS-D, State aR and EORTC are published elsewhere.

Statistics of both parts of the study

A reliability analysis of all 12 items of the ICS was carried out for all participants of study 1 with SPSS 15.0 to check for item-item and item-total-correlation of r ≥ 0.40 and a Cronbach's-α-reliability of r ≥ 0.70. The test-retest reliability was assessed with the 65 persons completing the questionnaire by Spearman's rank correlation. For all participants, we performed an orthogonal principal components analysis (rotation: varimax with Kaiser normalisation). We used the self-regulation questionnaire as a main convergence criterion because it's two dimensional scale is measuring the adaptive capacitiy. "Ability to Change Behaviour", and "Achieve Satisfaction and Well-Being" dealing with oncological disease. In addition, we performed a Spearman's rank correlation analysis for the whole sample, testing for associations between the ICS sum- and subscales on one hand and the assumed convergence criteria on the other. We assessed the discriminant validity by applying the Mann-Whitney U-test to check whether ICS sum- and subscales and self-regulation are differentiating between cancer and healthy controls, and by comparing both results. We estimated the responsiveness in measuring

1) chemotherapy sensitivity within the B and C group with the one-sided Wilcoxon's signed-rank test as well between first and second as second and third test-point.

## Results

### Part 1 of the study

#### Participants

59 consecutive patients with malignant conditions and 59 healthy controls were invited to participate in the study 1. In total, 114 persons agreed to participate (recruitment rate of 97%). From the 57 recruited cancer patients, we had 41 men and 16 women with a mean age of 59.3 years. Age and gender matching with the comparison group was successful (table 1).

Twenty-two patients had no metastases, 35 had a metastatic or generalized disease. The median KPI at the time of recruitment was 90%. The duration of the disease was on average 2.9 years. The mean haemoglobin (Hb) level was 12.1 g/dl (SD = 1.84). Further participant details are listed in table 2.

#### Analysis of reliability

All 12 items were checked: In the first step "when I felt warm I felt well" and "I had nightmares" were eliminated because of insufficient item-total correlation (0.20 and 0.32 respectively). The other 10 items fulfilled all reliability criteria:

Item-Item-correlations: r = 0.49, min – max = 0.25 – 0.84. The mean of item-variance is = 1.06 (min – max = 0.76 – 1.28). The corrected item-total correlation is:

rtr = 0.53 – 0.82. Cronbach's-α of the ICS sumscore rα = 0.91, retest-reliability rrt = 0.80 (p < 0.001) (table 4).

#### Principal component analysis

Primary factor analysis points to a two-principal-components model. The two-principal-components model (8 resp. 2 items) exhibits the attractive feature of unambiguousness in factor loading. Principal component 1 (inner resilience and coherence) is measured by 8 items (range: 8 – 40) explaining 44.2% of the variance, the second principal component (thermo coherence) is analysed by 2 items (2–10) which explains 23.0% of variance. The total scale explains a variance of 67.2%. In this model, the factors show a largely unambiguous item analysis pattern (table 4).

Cronbach's-α of the ICS inner resilience and coherence rα = 0.91, retest-reliability rrt = 0.74 (p < 0.001), and Cronbach's-α thermo coherence rα = 0.85, retest-reliability rrt = 0.54 (p < 0.001) (table 4).

The ICS significantly correlates with the concurrence criteria Trait aR, orthostatically-circulatory regulation, rest/activity regulation, anxiety and depression scores of the HADS-D, KPI, and with the convergence criteria self-regulation and it's subscales "achieve change in behaviour" and "achieve satisfaction" (r = 0.30 – 0.70, each with p = 0.001). Inner resilience and coherence correlates with the convergence criteria of r = 0.26–0.70 (p = 0.008), and thermo coherence of r = 0.19–0.32 (p = 0.04) (table 5). Of particular emphasis is the strong correlation between the "inner resilience and coherence" and the SRQ scale (ability to) "Achieve satisfaction" (r = 0.70), and the strong negative association with depression (r = 0.67) which is sound from a conceptual point of view.

**Table 5:** Correlation matrix studies 1 and 2 (first survey) of internal coherence, inner resilience and coherence, thermo-coherence with convergence criteria

	**Internal Coherence (ICS)**	**Inner Resilience & Coherence**	**Thermo Coherence**
Study 1: Inner Resilience and Coherence	.97*		
Study 1: Thermo Coherence	.63*	.50*	
Study 1: Self-regulation	.66*	.66*	.32*
Study 1: Achieve a change in behaviour	.52*	.52*	.32*
Study 1: Achieve satisfaction	.70*	.70*	.31*
Study 1: aR sum-scale	.41*	.37*	.27*
Study 1: aR orthostatic-circulatory	.30*	.26*	.19*
Study 1: aR rest/activity	.44*	.42*	.28*
Study 1: aR digestive	.09	.05	.09
Study 1: Karnofsky-Index	.53*	.50*	.32*
Study 1: HADS Anxiety	-.56*	-.56*	-.31*
Study 1: HADS Depression	-.63*	-.65*	-.33*
Study 1: less cold hands even in warmer months	.26*	.22*	.36*
Study 1: less perspiration	.29*	.29*	.32*
Study 1: less feeling cold	.34*	.25*	.22*

			

Study 2: EORTC Physical Functioning	.48*	.47*	.20
Study 2: EORTC Role Functioning	.39*	.36*	.23
Study 2: EORTC Emotional Functioning	.73*	.21	.71*
Study 2: EORTC Cognitive Functioning	.36*	.35*	.15
Study 2: EORTC Social Functioning	.31*	.26	.25
Study 2: EORTC Global Health	.66*	.58*	.47*
Study 2: EORTC Fatigue	-.34*	-.32*	-.31*
Study 2: EORTC Sleep Disturbances	**-**.20	-.19	-.10
Study 2: EORTC Nausea	-.18	-.27	-.12
Study 2: EORTC Pain	.35*	.40*	.11
Study 2: EORTC Dyspnea	.33*	.29	.44*
Study 2: EORTC Appetite loss	.53*	.48*	.34*
Study 2: EORTC Constipation	.11	.11	.19
Study 2: EORTC Diarrhea	.28	.18	.25
Study 2: EORTC Financial Difficulties	.25	.21	.21

* p < 0.05; correlations are presented bold.

The malignant group has a lower internal, inner resilience (both p < 0.001) and thermo coherence (p = 0.028) than the control group, whereas with self-regulation only "achieve satisfaction" (p = 0.012) shows a significant difference, whereas this does not hold for the subscales "achieve change in behaviour" and the total scale (p = 0.517, p = 0.079) (table 6).

**Table 6:** Mean values of ICS score of gender, age groups, cancer, breast cancer, colorectal cancer and healthy controls

	**Internal Coherence Scale**	**Inner Resilience and Coherence**	**Thermo Coherence**	**Self regulation**
**STUDY 1**				
**Gender**				
Female (71.9%)	36.52 (7.82)	28.67 (6.44)	7.91 (1.53)	4.05 (0.80)
Male (28.1%)	38.84 (7.17)	30.32 (5.91)	7.94 (1.46)	4.25 (0.65)

**Individuals**				
Healthy controls (SD)	**40.12 (5.12)**	**31.43 (4.48)**	**8.25 (1.30)**	4.27 (0.72)
Cancer (SD)	**34.23 (8.68)**	**26.80 (7.07)**	**7.60 (1.64)**	3.97 (0.78)

p-value *	**<0.001**	**<0.001**	**0.028**	0.079

**STUDY 2**				
Breast cancer group 1 T0	36.12 (7.64)	28.41 (6.54)	7.70 (2.28)	
Breast cancer group 1 T1	34.88 (7.53)	27.29 (6.25)	7.58 (1.97)	
Breast cancer group 1 T2	35.35 (6.38)	27.94 (5.99)	7.41 (1.97)	
Colorectal cancer T0	38.87 (6.49)	30.43 (5.81)	8.30 (1.94)	
Colorectal cancer T1	35.88 (9.36)	28.47 (7.79)	7.12 (2.33)	
Colorectal cancer T2	38.96 (6.75)	32.04 (5.44)	7.48 (2.27)	

p-value T0 vs T1 **	**0.026**	**0.043**	**0.020**	
p-value T1 vs T2**	**0.009**	**0.008**	0.268	

* Exact U-Test (2-side), ** Exact Wilcoxon-Test (1-side) used for pooled breast cancer and colorectal cancer and tests T0 (before chemotherapy) vs. T1 (1–5 days after third chemotherapy cycle) and T1 vs. T2 (4 – 8 weeks after the end of chemotherapy).

Significant differences (p < 0.05) are presented bold.

### Part 2 of the study

1) 18 patients who were consecutively surveyed for the B section were included in the study, with one patient refusing a third questionnaire. The questionnaires of the 17 remaining patients were available at all times and could be evaluated without curtailing validity.

2) 27 patients with CRC were surveyed for the C section before, during and after chemotherapy. 25 of these could be fully evaluated at all three stages.

ICS shows relations in varying degrees to quality of life as assessed with the EORTC (r = 0.08 – 0.71) and partly weak to moderate correlations to the symptom scales of the EORTC as fatigue or loss of appetite (= -0.53 – -0.28), but also partly no correlation at all such as with constipation etc. Of outstanding relevance is the strong correlation between "Inner Resilience and Coherence" and EORTC's "Emotional Functioning" (r = 0.73) and "Global Health" (r = 0.58) (table 5).

The pooled breast cancer group and CRC group showed a significant reduction of the ICS sum- and subscales during chemotherapy and a significant ICS increase after finishing chemotherapy (p < 0.044), with the exception of thermo coherence 4 to 8 weeks after chemotherapy (table 6). These results are consistent with the main results of the EORTC-QLQ C30 with a significant reduction of physical and cognitive functioning, and a significant increase of physical and role functioning and global health (table 7). The above mentioned results point towards a relevant correlation with quality of life and a scale sensitive to change during chemotherapy.

**Table 7:** Mean values of EORTC QLQ C30 functional scales of breast cancer and colorectal cancer

**STUDY 2**	**Quality of Life (EORTC)**					
	**physical**	**role**	**emotional**	**cognitive**	**social**	**Global**
Breast cancer group 1 T0	78.75 (26.47)	66.67 (30.21)	54.17 (25.89)	85.42 (19.12)	67.78 (30.52)	60.42 (21.62)
Breast cancer group 1 T1	72.94 (23.27)	55.88 (30.01)	50.00 (21.73)	77.08 (22.27)	56.67 (33.21)	51.56 (22.20)
Breast cancer group 1 T2	76.86 (20.43)	64.71 (33.27)	46.08 (23.59)	68.63 (29.39)	57.84 (33.39)	58.33 (26.19)
Colorectal cancer T0	77.22 (23.42)	58.67 (38.53)	60.33 (26.60)	80.67 (19.65)	62.32 (28.07)	57.00 (22.91)
Colorectal cancer T1	69.07 (25.16)	60.00 (35.35)	63.19 (24.19)	71.33 (24.31)	67.33 (32.80)	54.71 (23.14)
Colorectal cancer T2	75.83 (19.67)	78.26 (27.26)	71.01 (25.85)	79.17 (19.19)	76.09 (25.04)	63.04 (25.48)

p-value T0 vs T1 **	**0.008**	0.396	0.434	**0.013**	0.486	0.126
p-value T1 vs T2**	**0.024**	**0.012**	0.169	0.303	0.175	**0.016**

** Exact Wilcoxon-Test (1-side) used for pooled breast cancer and colorectal cancer and tests T0 (before chemotherapy) vs. T1 (1–5 days after third chemotherapy cycle) and T1 vs. T2 (4 – 8 weeks after the end of chemotherapy). Significant differences (p < 0.05) are presented bold.

## Discussion

We were able to develop a short inventory on internal coherence (ICS) with a 2-factor structure – with the factors 1) inner resilience and coherence and 2) thermo coherence – with good to very good internal consistencey [21] and a sufficient – good test-retest reliability. As the initial expert rating for item generation was based on a previous cancer patient symptoms list, we renounced designing the study further patient reports and interviews. The responsiveness of the ICS was tested with chemotherapy treatment:

As the decrease in quality of life, or the increase in adverse events (AE) during chemotherapy respectively is well known [22] chemotherapy sensitivity (responsiveness) in the pooled B and C group was documented. Moreover, compared to the SRQ according to Grossarth-Maticek, the ICS sum scale and subscales show a better sensitivity for tumour patients in study 1 [12]. During chemo- or radiotherapy, the 'locus of disease control' is completely externalised, and thus measures of "self-autonomy" should be low. Even after chemotherapy, a more depressive state of "self-competence" may persist in several patients, particularly those with a poorer prognosis. The scale "Inner resilience and coherence" in fact was strongly associated with emotional functioning and depression and thus is expected to be a more sensitive measure of the stressful situations, but it did not correlate with tumour stage in our data. In study 2, compared to the EORTC quality of life subscales with 42% significant test results, the ICS showed in 83% significant test results. Interestingly, the EORTC show above all chemotherapy sensitivity in physical, cognitive and role functioning and not in the scales being strongly correlated with the ICS, as emotional functioning and only at one point in the global health scale. This could be related to the individual self-management. Hence, the ICS as a scale capturing the individual skills of adaptation could be more sensitive for responsiveness as the fixed passive emotional functioning HRQOL-measure.

High ICS, including high inner resilience and coherence and thermo coherence level, are consistent with higher self-regulation, autonomic regulation, quality of life, lower depression and anxiety, along with better perception of health and quality of life (table 5), which emphasizes the salutogenetic significance. On the other hand, lower ICS points towards oncological morbidity, worse performance-index, more chemotherapeutical side effects and increase in symptoms, as well as reduced salutogenetic counter-regulation (table 4). Even if thermo coherence correlations coefficients to the convergence criteria are rather moderate until low, there is face validity that thermal comfort influence not only quality of life and a feeling of coherence [15]. There are further data showing that feeling cold and cold hands are well known distinctive side-effects under chemotherapy [23]. To what extent an accumulation of risk factors is involved remains to be clarified.

Antonovsky's Sense of Coherence Scale (SOC) is a reliable, valid and transculturally common instrument [24] which surveys long-term life orientation and beliefs, partly looking backwards, partly looking into the future, which reflect the understandability, importance and manageability of life events and stressors [8,9]. According to Antonovsky, SOC stabilises as a trait during adolescence [6]; however, new empirical data show changes following interventions and life events [10,25]. It has been very clearly shown that the focus of the SOC scale is not suitable for the capture of current, clinically relevant feeling and state of coherence for clinical questions in internal oncological patients, because of the very backward-looking, general style of questions and the face validity [24].

Our ICS scale refers to the previous week and queries perception of comfort and health; questions on inner balance, particularly with respect to the ability of problem solving, security and inner congruence and resilience in terms of importance and manageability, and two additional items addressing perceptions of coldness/chillness and warmth ('thermo coherence'). In the moderate correlation of the two items of the thermo coherence subscale with the inner resilience and coherence subscale and self-regulation, we recognized a first confirmation of our hypothesis that thermoregulation and coherence in cancer patients could be related. Particularly, because tolerance of cold is moderately associated with less perspiration and high level thermo coherence which could be linked with the thermo-regulatory threshold between perspiration and vasoconstriction is depending on circadian rhythm, vigilance, personality coherence and gender [26]. Hence, we decided to let both subscales integrated in the ICS scale, but this factor structure should be controlled with a larger number of patients. The recorded higher sensitivity of the ICS to differentiate between cancer patients and healthy controls as compared to self-regulation indicates a higher cancer sensitivity. To what extent the postulated cancer and treatment sensitivity of the ICS in contrast to the SOC questionnaire is cancer specific, remains to be verified in further studies.

A limitation of SOC is that the three-factor structure, as postulated by Antonovsky, could not be reproduced and factor-loading pattern are varying significantly in the different languages ([27,28]. Moreover, the scale was mainly used and validated sociologically, socio-medically and psychiatrically [24]. As most existing studies are cross-sectional studies, Antonovsky's basic assumption that the SOC is a principal determinant of health in terms of an ability to maintain particularly mental health despite of significant 'stressors', has not yet been sufficiently verified. Even if the data generated in the process show moderate to strong negative correlation between SOC on the one hand and anxiety, depression and distress on the other hand, as well as positive correlations to quality of life and subjectively gauged health, in particular mental health, there is still controversial discussion if SOC is an independent predictor for anxiety and depression or depends on un-existence of anxiety and depression [29,27]. In earlier prospective studies, the results were initially inconsistent [30], but now there are studies which seem to have a prognostic implication, amongst others, for cardiovascular mortality [31], for the risk of stroke [32], as well as a 46% increased prevalence of Diabetes mellitus Type 2 over a period of 17 years was associated with weak SOC [33].

Recently a large Finnish study showed a marginally higher 12-year cancer incidence with lower SOC in elderly [34]. The EPIC-Norfolk-Study has also shown that lower SOC is associated with an increased global mortality and cancer mortality [31]. These results match a cross-sectional study from Hawaii which systematically examined oncological patients with unusually long survival rates which did not differ from a control group with regards to their quality of life but only with regards to their SOC levels [7]. These results are concordant with higher life expectancy of cancer patients with higher self-regulation level as compared to matched pairs – with an additional positive effect discovered amongst those receiving complementary mistletoe therapy [35]. In fact, we can confirm strong correlations between the SRQ and the ICS and inner resilience and coherence scale and moderate with the thermo coherence scale. Despite these interesting results which indicate a positive influence of changing behaviour to reach goal and well-being orientated lifestyle in the context of salutogenesis on cancer incidence and progression, the importance of the SOC in large prospective studies has not yet been conclusively validated and requires further validation. The clinical and prospective importance of the new ICS remains to be clarified in future studies, but we intend to use it to capture more appropriately inner attitudes in palliative and disease-free cancer patients under chemo or mistletoe therapy, and to measure the influence of psychosocial support with this cancer specific individual skills of adaptation detecting tool.

There are significant limitations in our study in the lack of parallel recording of the SOC scale in the first and second part of the study. In unpublished data of our group, ICS correlates in cancer patients under treatment moderately with the SOC scales (comprehensibility: r = 0.50, meaningfulness: r = 0.49, manageability: r = 0.47 (all p < 0.05) indicating clearly the difference of the SOC, being a trait-marker and ICS as a more clinical tool referring to the last week. It must also be mentioned that the ten items of the ICS only query comprehensibility indirectly. In the second part of the study, we used a 1-side Wilcoxon test for measuring chemotherapy sensitivity of the scale because of clear hypothesis that ICS scores will decrease during chemotherapy and raise afterwards, and to minimize alpha-error due to the small patient number and because of concomitant mistletoe therapy already reducing side-effects of chemotherapy [36,37]. Hence, we accepted a potential over-estimation of the beta-error. The correlative associations between ICS and its subscales, anxiety and depression scale are rather less strong than with the SOC; Geyer [38] discusses to what extent the SOC would be largely dependent on symptoms of anxiety and depression (♂: r = 0.42, ♀: r = 0.53 – 0.85). Nevertheless, further studies should clarify if ICS is more than simply the absence of anxiety and depression.

## Conclusion

The development of an Internal Coherence Scale (ICS) was achieved with good to very good reliability. First validity tests showed good convergence (Self-regulation), concurrence and discriminant validity between cancer patients and healthy control and confirmed a good responsiveness of the ICS during cancer treatment. Therefore, ICS could be an interesting instrument to capture the feeling of inner coherence and resilience among cancer patients. Further studies to confirm the scale structure and clinical relevance are required.

## Competing interests

The authors declare that they have no competing interests.

## Authors' contributions

MK, MG, HBvL, FS, MR designed the questionnaire, MK, MR performed statistical analysis, MK, AB, HM drafted the manuscript; HBvL, MG, GF participated in editing the manuscript. All authors read and approved the final manuscript.
